# Reconceptualizing English language learning resilience from positive psychology: scale development and psychometric validation

**DOI:** 10.3389/fpsyg.2025.1685688

**Published:** 2025-11-28

**Authors:** Ting Yin, Jiexin Chen, Zhiqing Lin

**Affiliations:** 1 School of Foreign Languages, Guangdong Polytechnic Normal University, Guangzhou, China; 2 School of Foreign Languages, Neusoft Institute Guangdong, Foshan, China

**Keywords:** positive psychology, resilience scale, measurement invariance, psychometric validation, language learning

## Abstract

**Background:**

Resilience, a central construct in positive psychology, plays a crucial role in helping language learners cope with and grow from the challenges inherent in English language learning. However, few systematically validated instruments are available to assess resilience within this specific context. To address this gap, the present study developed and validated the English Language Learning Resilience Scale.

**Methods:**

Data were collected from 617 Chinese university students. The internal structure of the scale was examined through exploratory and confirmatory factor analyses, followed by measurement invariance testing. Additionally, the graded response model was applied to evaluate the psychometric properties of individual items.

**Results:**

Results of factor analysis identified five protective factors—value, perseverance, emotion regulation, social support, and self-efficacy—demonstrating satisfactory convergent and discriminant validity. Furthermore, the scale showed scalar measurement invariance across gender, age, and academic discipline, indicating its structural stability across diverse subgroups. Complementing these findings, item response theory analyses provided additional evidence of strong item discrimination, appropriate difficulty parameters, and high measurement precision.

**Conclusion:**

Theoretically, the study reconceptualizes resilience in language learning through the lens of positive psychology, offering a more nuanced understanding of its dimensions and functions. Practically, the scale offers educators a diagnostic tool for identifying learners with lower resilience and implementing targeted interventions to strengthen their capacity to adapt, persist, and thrive in the face of linguistic challenges, thereby promoting both their language learning success and overall psychological well-being.

## 1 Introduction

Positive psychology has become increasingly integrated into second language research and has experienced substantial growth in the fields of second language acquisition (SLA) and language teaching in recent years (MacIntyre et al., 2019; Dewaele et al., 2019; Wang et al., 2021). As a fundamental construct within positive psychology, resilience has been recognized as a critical psychological resource, which plays a key role in shaping learners’ emotional, motivational, and cognitive responses in second language learning (Liaqat et al., 2025; Chen et al., 2025).

For example, empirical studies indicate that resilience can mitigate English as a Foreign Language (EFL) learners’ negative emotions, such as anxiety, stress, and burnout (Shen, 2022; Wang et al., 2024). Beyond emotion regulation, resilience also fosters greater academic buoyancy, helping students to maintain consistent engagement in the learning process (Li, 2022). In addition, resilience itself serves as a motivating force, enabling learners to confront challenges and persevere in their English learning (Zhang, 2022; Danesh and Shahnazari, 2020). Moreover, it enhances students’ adaptive capacities in self-regulated learning, allowing them to navigate potential difficulties more effectively (Wang et al., 2021). Taken together, these findings underscore the central role of resilience in fostering positive adaptation and sustained engagement in EFL learning (Shi and Gao, 2025).

While scholarly interest in resilience has substantially increased (e.g., Ang et al., 2022; Cui et al., 2023; Nooripour et al., 2022), the conceptualization and measurement of resilience in language learning contexts remain underexplored. Existing general resilience instruments (DeSimone et al., 2017; Surzykiewicz et al., 2018) may not perform consistently across different learning environments, as their psychometric properties are not specifically validated for the unique challenges of language learning. This contextual specificity highlights the importance of developing and validating resilience scales tailored for EFL learners, addressing a critical gap in the current literature. Moreover, the only existing resilience scale for English language learning (Guo and Li, 2022) is limited by insufficient conceptualization of the resilience construct and has not been rigorously calibrated using Item Response Theory (IRT) models. In addition, the generalizability of previous resilience scales in SLA across age, gender, and academic major has not been systematically investigated, particularly in the EFL context. Collectively, these limitations highlight the need for a theoretically grounded and empirically validated resilience scale for EFL learners in the Chinese context.

To address these gaps, the present study seeks to develop and psychometrically validate an English language learning resilience scale. The scale development process involves conducting exploratory factor analysis (EFA) to identify the latent factor structure, followed by confirmatory factor analysis (CFA) to test the hypothesized structure derived from EFA and to assess model fit. Furthermore, measurement invariance testing will be implemented to examine the scale’s equivalence across diverse learner subgroups. To strengthen the psychometric validity evidence, this study will employ graded response modeling to assess the scale’s measurement properties comprehensively.

The development of this scale carries important theoretical and practical implications for English language education. Theoretically, it can deepen our understanding of the theoretical reconceptualization of resilience in foreign language learning from a positive psychology perspective. From a practical perspective, the scale will serve as a valuable diagnostic tool for educators to identify at-risk learners and implement positive psychology-informed support mechanisms.

## 2 Literature review

### 2.1 Resilience in language learning from a positive psychology perspective

Resilience, a cornerstone construct in positive psychology, has emerged as a significant focus in language education research. The advent of positive psychology in applied linguistics has particularly underscored resilience’s pivotal role in learning processes (Wang et al., 2021). Scholars have increasingly recognized two interrelated dimensions through which resilience shapes language learning: its capacity to regulate learners’ emotions in the face of challenges, and its function as a form of academic buoyancy that supports sustained performance and adaptation (Chu et al., 2024).

On the one hand, resilience serves as a critical emotional regulator, empowering learners to navigate challenging learning situations and recover from daily academic setbacks (Zarrinabadi et al., 2022). Empirical evidence positions resilience as a key mediator between language learners’ emotion regulation strategies and their capacity for autonomous learning (Shafiee Rad and Jafarpour, 2023). Notably, learners demonstrating higher resilience levels exhibit stronger self-directed learning motivation and enhanced psychological well-being (Wang and Liu, 2022), suggesting its fundamental role in sustaining long-term language learning engagement.

On the other hand, resilience operates as academic buoyancy—a dynamic protective factor that positively correlates with learning outcomes (Martin and Marsh, 2008). Research consistently indicates that resilient learners adapt more effectively to academic environments, resulting in superior performance metrics (Putwain et al., 2013). Within L2 contexts specifically, resilience has been shown to significantly predict both learning motivation and ultimate proficiency attainment (Kim and Kim, 2016). Moreover, resilient learners exhibit enhanced cognitive engagement, performing more effectively in demanding tasks such as reading comprehension (Kamali and Fahim, 2011) and demonstrating greater willingness to participate in interactive activities like storytelling (Nguyen et al., 2015). This body of research collectively characterizes resilient language learners as simultaneously more engaged and psychologically fulfilled participants in the learning process (Zarrinabadi et al., 2022), establishing resilience as a crucial determinant of both academic success and emotional well-being in SLA contexts.

In sum, resilience emerges as a multifaceted psychological resource that not only enables learners to manage affective challenges and academic adversities but also fosters greater engagement and long-term success in language learning (Zhang, 2022). Its dual function—as both an emotional buffer and a cognitive enhancer—underscores its indispensable role in shaping learners’ motivation, behavior, and outcomes.

### 2.2 Conceptualization of resilience from multiple perspectives

Resilience is a concept originating from the Latin term *resilire*, which refers to the ability to spring back into shape after bending or stretching (Windle, 2011). This concept was first systematized by Holling (1973) in ecology and was later introduced into developmental and clinical psychology (Richardson, 2002).

In the research related to these two disciplines, resilience is fundamentally defined as the dynamic capacity to recover from adversity, adapt to challenges, and achieve positive adjustment (Mancini and Bonanno, 2006; Aldosari and Alsager, 2023). For instance, DeSimone et al. (2017) defined resilience as a five-dimensional construct comprising optimism, social support, emotion regulation, self-efficacy, and adaptability. Similarly, Hjemdal et al. (2015) conceptualized resilience as a six-factor model, including perception of self, planned future, social competence, structured style, family cohesion, and social resources. Moreover, Reivich and Shatte (2002) identified seven core competencies of resilient individuals: (1) emotion regulation, (2) impulse control, (3) causal analysis, (4) empathy, (5) realistic optimism, (6) self-efficacy, and (7) proactive help-seeking behavior. These studies tend to conceptualize resilience as the competence to cope with challenges and bounce back with external support.

The concept of resilience has also been defined in positive and educational psychology (Chu et al., 2024). In this field, resilience is conceptualized as learners’ capacity to overcome academic adversities that threaten educational progress (Martin, 2013). For example, Martin and Marsh (2008) operationalized it as students’ adaptive competence to implement effective strategies when facing challenging learning situations, with particular emphasis on stress management in educational contexts. Cassidy (2016) defined academic resilience as a three-factor construct, including (1) perseverance, (2) reflective and adaptive help-seeking, and (3) negative affect and emotional response. Similarly, Smith et al. (2010) conceptualized resilience in positive psychology as an individual’s capacity to recover from stress, highlighting the adaptive processes through which people confront challenging situations.

As seen above, resilience has been conceptualized as a multidimensional construct in both developmental, clinical, educational, and positive psychology. These studies reveal resilience as a multifaceted psychological concept whose operationalization varies across domains, yet consistently reflects adaptive functioning in challenging contexts.

### 2.3 Defining the resilience constructs for the resilience scale

Building on its conceptualization across various disciplines of psychology in the previous section, resilience is understood as a multidimensional construct comprising five interrelated factors: value, perseverance, emotional regulation, social support, and self-efficacy. These factors align with previous process-oriented approaches to defining resilience (see Chu et al., 2024; Fletcher and Sarkar, 2013), as they capture how learners initiate, sustain, and recover their engagement in English learning when facing challenges.

Each dimension is further supported by relevant psychological theories, ensuring a strong theoretical foundation. To be specific, the first dimension, value, reflects learners’ perceived importance of succeeding in English learning and is underpinned by Expectancy-Value Theory, which suggests that motivation is driven by the subjective significance attached to learning outcomes (Wigfield, 1994). Perseverance, the second dimension, denotes learners’ tenacity and sustained effort in the face of difficulties, consistent with Psychological Capital Theory, which emphasizes that personal strengths play a key role in maintaining engagement over time (Luthans et al., 2007). Emotional regulation reflects learners’ ability to manage negative emotions and setbacks during the learning process and is informed by Stress and Coping Theory, which proposes that effective coping strategies enable individuals to adapt to stressors (Folkman, 1984). Social support reflects learners’ access to external resources that help them navigate difficulties and is derived from Social Connectedness Theory, which holds that supportive interpersonal relationships enhance individuals’ ability to cope with challenges (Lee and Robbins, 1995). Finally, self-efficacy reflects learners’ confidence in their ability to achieve future learning goals and is based on the Self-Efficacy Theory, which asserts that individuals’ beliefs in their capabilities enable proactive engagement and persistence in the face of obstacles (Bandura, 1977).

Collectively, these five factors provide a comprehensive framework for reconceptualizing EFL learning resilience, encompassing motivational (value), behavioral (perseverance), cognitive (self-efficacy), emotional (emotion regulation), and interpersonal (social support) dimensions of positive psychology. Within this perspective, learners are viewed as active agents who draw upon both internal strengths and external resources to activate, maintain, and rebuild their engagement in language learning.

### 2.4 Existing resilience scales for language learning and research gaps

Some previous scholars have developed resilience scales across various social science disciplines. Notable examples include the mathematical resilience scale (Ricketts et al., 2015), the resilience scale for nurses (Adıbelli et al., 2021), the physical-related resilience scale for athletic adolescent girls (Nooripour et al., 2022), the academic resilience scale (Cassidy, 2016), the language teacher resilience scale (Liu et al., 2024), and the language learning resilience scale (Guo and Li, 2022). These studies make important contributions to the development and validation of resilience scales and provide useful tools for the measurement of resilience in different contexts.

However, according to the systematic review on resilience scale conducted by Windle et al. (2011) and Salisu and Hashim (2017), three critical limitations warrant attention: (1) construct representation adequacy, (2) contextual specificity, and (3) validation comprehensiveness.

First, existing scales often suffer from construct underrepresentation. For instance, the most relevant English language learning resilience scale was developed by Guo and Li (2022), who investigated three aspects of resilience (ego, metacognitive, and social aspects of resilience), making a significant contribution to the development and validation of resilience measures. However, this scale did not account for other important constructs of resilience, such as emotional regulation and perseverance. This narrow theoretical foundation fails to capture the multidimensional nature of resilience, potentially overlooking key components identified in broader psychological frameworks. As a result, such scales may lack sufficient breadth to fully represent the constructs of resilience in positive psychology.

Second, contextual misalignment poses a significant concern. Many of the existing scales originate from general or domain-specific settings rather than being tailored to language learning environments. This mismatch raises questions about their ecological validity for EFL learners, which underscores the need to examine whether the scale performs consistently across different demographic groups, such as gender, age, and academic discipline.

Third, prior research on English language learning resilience has been limited in terms of comprehensive psychometric validation using advanced techniques. To date, the only existing scale in this context, developed by Guo and Li (2022), has not undergone thorough calibration with psychometric models. Furthermore, few studies have applied item-level analyses, such as the graded response model in IRT models. These analyses generate detailed parameters, including discrimination and threshold estimates, which provide deeper insights into scale functioning (Edelen and Reeve, 2007; Huang et al., 2023). Such item-level information is particularly valuable for resilience measurement (Gao and Chen, 2025), where understanding item characteristics is essential for both theoretical precision and practical application. Consequently, the underutilization of IRT-based models underscores a critical gap in existing resilience research, as these models enable more diagnostic, scalable, and generalizable measurement (Gempp and Gonzalez-Carrasco, 2025).

In summary, despite valuable prior efforts, limitations in construct coverage, contextual relevance, and analytical rigor point to the need for a new, EFL-specific resilience scale. Given the pivotal role of resilience in language learning, the current study aims to develop and validate a comprehensive resilience scale for foreign language learning, guided by the following research questions:

*RQ1:* What is the factor structure of the English language learning resilience scale?

*RQ2:* Does the resilience scale demonstrate measurement invariance across gender, age, and academic disciplines?

*RQ3:* What insights does the graded response model provide regarding the validity of the resilience scale?

## 3 Methodology

### 3.1 Item pooling for scale development

Based on the five subconstructs reviewed in section 2.3, we developed corresponding items to build the initial item pool. For the value dimension, five items (Items 1, 6, 11, 16, 21) were adapted from the mathematical resilience scale by Kooken et al. (2015). For perseverance, five items (items 2, 7, 12, 17, 22) were developed with reference to Teimouri et al. (2020). The emotion regulation dimension included six items (items 3, 8, 13, 18, 23, 26), adapted from the emotion regulation questionnaire by Gratz and Roemer (2004). For social support, five items (items 4, 9, 14, 19, 24) were constructed based on findings from Jin and Dewaele (2018). Lastly, five items (items 5, 10, 15, 20, 25) for self-efficacy were adapted from the resilience scale developed by DeSimone et al. (2017) in health psychology. All the information was summarized in Table 1.

**Table 1:** The process of item pooling.

Subconstruct	Number	Item numbers
Value	5	1, 6, 11, 16, 21
Perseverance	5	2, 7, 12, 17, 22
Emotion regulation	6	3, 8, 13, 18, 23, 26
Social support	5	4, 9, 14, 19, 24
Self-efficacy	5	5, 10, 15, 20, 25

To ensure content validity and clarity, three experienced college English teachers were invited to evaluate the appropriateness and quality of each item. Additionally, five students were asked to assess whether the items were clear and understandable, to identify any potential ambiguities or comprehension issues prior to the formal administration of the questionnaire. The initial questionnaire was developed in Chinese and subsequently translated into English by the author. The English version was then back-translated by two experts in English–Chinese translation to ensure equivalence between English and Chinese.

### 3.2 Data collection and cleaning

Ethical approval was obtained from the universities of the first and third authors of this study. Before administering the questionnaire, all participants were verbally informed about its purpose. They were also assured that the collected data would be anonymized and used solely for research purposes. Convenient sampling was used in this study. Participants were recruited from two different types of universities in China: one with a focus on engineering and the other on the humanities. A total of 690 university students initially completed the questionnaire, which was distributed via the Wen Juanxing online platform.

Before conducting data analysis, we performed data cleaning because careless responses could compromise the accuracy of scale development and validation (Hou et al., 2025). Two primary indicators were employed to detect potential insufficient effort responses: total response time and longstring index. The longstring index measures the number of consecutive identical responses. High values on this index may indicate inattentive responding, as participants who do not engage carefully with the questionnaire often select the same option repeatedly. Similarly, unusually short total response times may signal insufficient effort, reflecting participants’ tendency to complete the survey too quickly without adequately considering each item.

Following the recommendations of Wang et al. (2025), we combined multiple indicators to identify potential careless responses, as relying on a single criterion might produce false positives. Response times were automatically recorded by the Wenjuanxing platform used for questionnaire administration, and the longstring index was calculated using the careless R package (version 1.2.2).

However, there are no universally accepted thresholds for response times or longstring values, as the number of items varies across scales. Therefore, following the practice of previous research on data cleaning in scale development and validation (Hou et al., 2025), we excluded cases that simultaneously met both criteria—total response times more than two standard deviations below the sample mean and longstring index values exceeding twice the sample mean—as such patterns were likely indicative of insufficient effort responding. This dual-criterion approach effectively reduces misclassification and enhances the robustness of subsequent psychometric analyses.

After data cleaning, 617 valid responses were retained for subsequent analysis. These 617 participants ranged in age from 17 to 26 years, with a mean age of 20.14. 290 (47%) were male and 327 (53%) were female. In terms of academic majors, 261 students (42.3%) were majoring in engineering-related subjects, while 356 students (57.7%) were studying humanities-related disciplines. Regarding their year of study, 290 were first-year students, 118 were in their second year, 186 were in their third year, and 23 were in their fourth year.

### 3.3 Data analysis

Before data analysis, data screening was conducted. Skewness and kurtosis statistics were calculated to assess normality. The results indicated that all items followed approximately normal distributions.

To address RQ1, both EFA and CFA were conducted. To ensure the validity of these analyses, the full dataset (*n* = 617) was randomly divided into two equal subsamples, with one subsample used for EFA and the other for CFA. Initially, all 26 items were entered into the EFA. During this preliminary analysis, Item 18 was found to distort the factor structure, likely due to its negative wording. Following the scale development guidelines established by DeVellis and Thorpe (2021), this item was removed, resulting in a total of 25 items retained for EFA.

An iterative EFA approach was then adopted to explore the underlying factor structure and select appropriate items. Using the first subsample (*n* = 308), the 25 items were analyzed in SPSS (version 25) with principal axis factoring for extraction and equamax rotation. Three criteria guided item evaluation and selection: (1) items with misloadings were removed; (2) items with significant cross-loadings were excluded; and (3) items with low factor loadings were eliminated. Accordingly, Items 3, 7, 8, and 24 were deleted due to misloadings; Items 4, 20, 22, and 25 were removed because of cross-loadings; and Item 21 was deleted due to a relatively low loading within its construct (value). In total, nine items were removed, reducing the scale from 25 to 16 items. The refined 16-item scale was then subjected to another EFA.

Prior to analysis based on 16 items, the Kaiser-Meyer-Olkin (KMO) test was performed to assess sampling adequacy, yielding a value of 0.908, which indicates excellent suitability for factor analysis. Bartlett’s test of sphericity was significant (χ^2^ = 3070.400, df = 120, *p* < 0.001), confirming that correlations among items were appropriate for factor extraction. At this stage, all factor loadings exceeded 0.40, and no cross-loading or misloading items were identified. Detailed results are reported in the Results section.

Based on the results of the EFA, a confirmatory factor analysis (CFA) was subsequently conducted on the second subsample (*n* = 309) using SmartPLS-4 (version 4.1.0.9) to validate the factor structure identified in the EFA. During this process, we compared the fit of alternative models to determine the most appropriate factor structure. Convergent and discriminant validity were also assessed using SmartPLS-4. Specifically, convergent validity was evaluated via Average Variance Extracted (AVE) and factor loadings, following the practices of previous studies (Zhao et al., 2025; Liu and Chu, 2022). Discriminant validity was examined using the Heterotrait-Monotrait ratio (HTMT), which has been recommended as a more robust method than the Fornell-Larcker criterion (Henseler et al., 2015).

To address RQ2, we first divided participants into subgroups based on age and academic major for measurement invariance testing. Specifically, participants aged 17–19 were classified as group one (*n* = 319), while those aged 20–26 were classified as group two (*n* = 357). This grouping was chosen because it yielded the most balanced sample sizes between the two age groups, thereby ensuring comparability in subsequent analyses. In terms of academic discipline, students majoring in engineering-related fields were categorized as group A (*n* = 282), and those in humanities-related disciplines were categorized as group B (*n* = 394). Moreover, measurement invariance across subgroups (gender, age, and academic major) was examined through multiple-group CFA using lavaan packages in R (version 0.6–20). Three levels of invariance were tested: configural, metric (weak), and scalar (strong) invariance (Putnick and Bornstein, 2016).

To address RQ3, we employed the mirt package (version 1.35.1) to estimate item-level parameters under the graded response model based on the responses of 617 participants, including item discrimination and threshold (difficulty) parameters. Additionally, item information functions and measurement precision were analyzed to evaluate the diagnostic quality of each item and to provide further evidence of scale validity from an IRT perspective. All R-based analyses were implemented in RStudio (version 4.2.2). To promote transparency and adherence to reporting standards, a checklist based on the Journal Article Reporting Standards for Quantitative Research is provided in Appendix.

## 4 Results

### 4.1 Result of EFA and CFA

**Figure 1:**
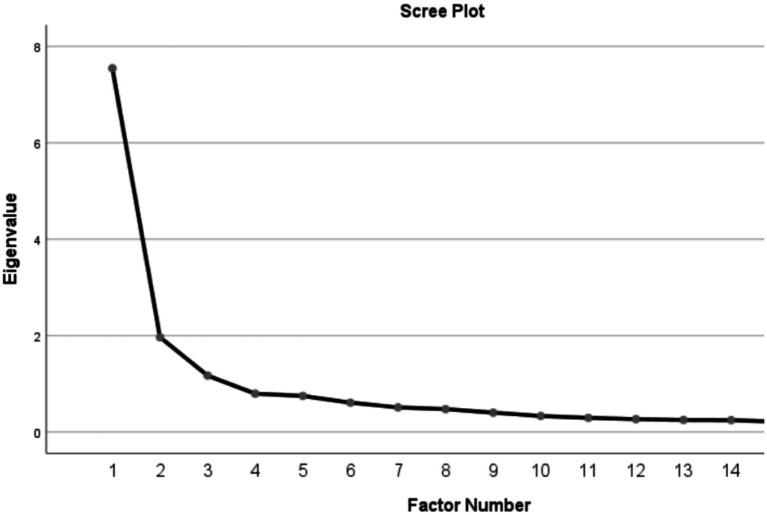
Scree plot in EFA based on the 16 items.

The scree plot (see Figure 1) during EFA supported the number of five factors, accounting for a total of 66.028% of the variance. The variance explained by each factor was 15.638% (value), 12.205% (perseverance), 13.550% (emotion regulation), 14.501% (social support), and 10.135% (self-efficacy), respectively. These results demonstrate the distinctiveness of the five resilience constructs in the English language learning scale. Generally, factor loadings in EFA can be interpreted as low (<0.4), acceptable (0.4–0.6), and satisfactory (>0.6) (Kline, 2014). As shown in Table 2, the factor loadings for all items within the five constructs exceeded the standardized thresholds. Specifically, for the value dimension, items 1, 6, 11, and 16 all demonstrated loadings above 0.6, indicating satisfactory construct validity. The factor loadings for the perseverance items (2, 12, and 17) were within the acceptable range, while the items for the emotional regulation construct (13, 23, and 26) exhibited satisfactory loadings. For the social support construct, items 9, 14, and 19 showed very high loadings, reaching 0.7, suggesting they were well designed to measure the intended construct. The self-efficacy items (5, 10, and 15) also displayed acceptable loadings. Overall, the EFA results indicate that all retained items were appropriate and effective for assessing resilience in English language learning.

**Table 2:** Results of EFA based on the retained 16 items.

Factors	1	2	3	4	5
Factor 1: Value					
Item 1. Learning English is beneficial for my future development.	0.772				
Item 6. No matter what job I pursue, having a good command of English will be advantageous.	0.71				
Item 11. Proficiency in English can help me achieve my goals.	0.71				
Item 16. Learning English well can help us better understand our professional knowledge.	0.62				
Factor 2: Perseverance					
Item 2. I can work hard to overcome obstacles in learning English.		0.452			
Item 12. I am diligent and serious in learning English.		0.807			
Item 17. I can stick to my English learning plans.		0.565			
Factor 3: Emotion regulation					
Item 13. I can manage the negative emotions that arise during English learning.			0.612		
Item 23. Negative emotions during English learning will not affect my learning progress.			0.683		
Item 26. I can regulate the negative emotions generated in the process of learning English.			0.788		
Factor 4: Social Support					
Item 9. I ask my roommates for help when I have problems with English.				0.733	
Item 14. I turn to my classmates when encountering difficult points in English.				0.743	
Item 19. I ask close friends for help with English difficulties.				0.874	
Factor 5: Self-efficacy					
Item 5. I believe that with continuous effort, I can improve my English proficiency.					0.449
Item 10. Everyone can make progress in learning English.					0.624
Item 15. I believe I can learn English well.					0.516

**Figure 2:**
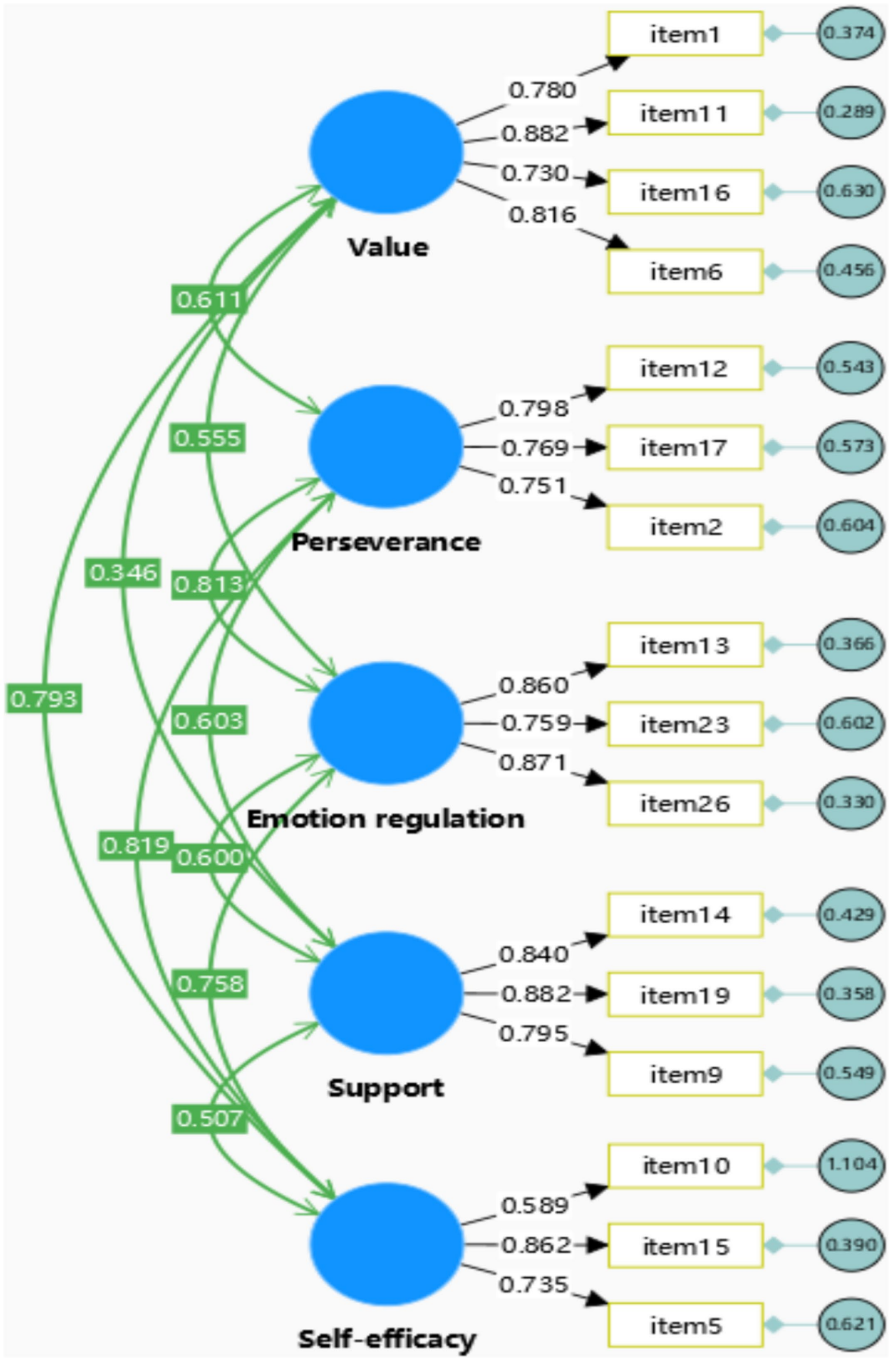
CFA model illustrating the factor-level structure of resilience.

Based on the results of the EFA, a CFA was subsequently conducted to confirm the structure of resilience. After inspecting the factor loadings of items and the model fit, 16 items were retained in the CFA model, which were illustrated in Figure 2. In general, the factor loadings of the items associated with the five subconstructs of resilience exceeded 0.70 (excluding item 10), indicating strong support for each construct.

In addition, the five-factor structure of resilience was further supported by the comparative analysis of model fit indices across the five CFA models. According to the interpretation guidelines proposed by Hu and Bentler (1999), values of Tucker–Lewis index (TLI) (> 0.900), Comparative Fit Index (CFI) (> 0.900), Root Mean Square Error of Approximation (RMSEA) (< 0.080), and Standardized Root Mean Square Residual (SRMR) (<0.080) indicate a good model fit.

As shown in Table 3, only the five-factor model demonstrated satisfactory fit indices, including CFI, TLI, RMSEA, and SRMR, whereas the fit indices of the other four models fell below the acceptable thresholds. Moreover, we observed that model fit steadily improved as the number of factors increased from one to five, suggesting that these five factors are distinct and meaningfully contribute to the construct. This pattern provides further support for the five-factor structure of the resilience scale developed in the present study.

**Table 3:** Model fit statistics of five CFA models.

Models	χ^2^	df	χ^2^/df	TLI	CFI	RMSEA	90% CI	SRMR
One-factor	1038.032	104.000	9.981	0.644	0.691	0.174	[0.161, 0.180]	0.171
Two-factor	954.386	103.000	9.266	0.672	0.718	0.164	[0.154, 0.173]	0.110
Three-factor	798.700	101.000	7.908	0.726	0.769	0.150	[0.140, 0.160]	0.099
Four-factor	580.147	98.000	5.920	0.802	0.841	0.126	[0.116, 0.136]	0.080
Five-factor	240.480	94.000	2.558	0.938	0.952	0.071	[0.060, 0.082]	0.039

One-factor model: All 16 items were loaded on a single factor. Two-factor model: (1) value and perseverance; (2) emotion regulation, social support, and self-efficacy. Three-factor model: (1) value and perseverance; (2) emotion regulation and social support; (3) self-efficacy. Four-factor model: (1) value; (2) perseverance; (3) emotion regulation; (4) social support and self-efficacy. Five-factor model: five distinct but correlated factors—value, perseverance, emotion regulation, social support, and self-efficacy.

Additionally, the five-factor model exhibited satisfactory convergent and discriminant validity. Following the interpretation guidelines proposed by previous studies (Sun and Shi, 2022; Cheung et al., 2023), convergent validity is supported when the average variance extracted (AVE) exceeds 0.50 and the standardized factor loadings exceed 0.70. As shown in Figure 2, all items—except item 10—demonstrated loadings above 0.70, and the AVE values for all five factors were greater than 0.50 (see Table 4), confirming adequate convergent validity. Furthermore, the heterotrait–monotrait (HTMT) ratios reported in Table 5 were all below 0.85, which meets the criterion suggested by Henseler et al. (2015) for discriminant validity. Collectively, these findings provide strong evidence for both the convergent and discriminant validity of the resilience scale.

**Table 4:** Reliability and convergent validity of the resilience scale.

Factors	Cronbach’s alpha (unstandardized)	Composite reliability (rho_c)	AVE
Regulation	0.869	0.870	0.691
Perseverance	0.815	0.817	0.598
Self-efficacy	0.766	0.772	0.543
Support	0.875	0.878	0.705
Value	0.876	0.879	0.647

AVE: average variance extracted.

**Table 5:** Discriminant validity of resilience scale based on HTMT.

Factors	Regulation	Perseverance	Self-efficacy	Support	Value
Regulation					
Perseverance	0.819				
Self-efficacy	0.738	0.814			
Support	0.599	0.616	0.530		
Value	0.543	0.619	0.812	0.350	

HTMT: Heterotrait-monotrait ratio.

### 4.2 Result of measurement invariance

Measurement invariance was assessed across three grouping variables: gender, age, and major. To begin with, the result shows that the configural model in these three groups shows good model fit, which lays the foundation for subsequent analysis. Specifically, metric invariance is considered to be supported when the change in ΔCFI is less than 0.010, the change in ΔRMSEA is less than 0.015, and the change in ΔSRMR is less than 0.030 (Botes et al., 2022). Scalar invariance requires more stringent criteria: ΔCFI < 0.010, ΔRMSEA < 0.015, and ΔSRMR < 0.010 (Chen, 2007; Maes et al., 2014).

As presented in Table 6, the differences in fit indices between configural and metric models across gender, age, and major all met the recommended thresholds (ΔCFI = −0.001 to −0.003; ΔRMSEA = −0.001 to −0.002; ΔSRMR = 0.001 to 0.010), supporting metric (weak) invariance across all three groups. Similarly, the changes between metric and scalar models also met the criteria for scalar invariance, with ΔCFI values below 0.010, ΔRMSEA changes within ±0.001, and ΔSRMR values below the 0.010 threshold. Therefore, the results demonstrate that scalar (strong) invariance was also established across gender, age, and major. These findings suggest that the resilience scale operates equivalently across different demographic groups, allowing for meaningful and valid comparisons between subgroups.

**Table 6:** Measurement invariance across different genders, ages, and majors.

Invariance testing	Model	χ^2^	df	χ^2^*/*df	*p*	CFI	RMSEA and 90% CI	SRMR	△CFI	△RMSEA	△SRMR
Invariance across											
genders	Configural	392.750	160.000	2.455	0.000	0.950	0.077 [0.069, 0.085]	0.039			
	Metric	405.100	170.000	2.383	0.000	0.949	0.075 [0.067, 0.083]	0.043	−0.001	−0.002	0.004
	Scalar	430.740	180.000	2.393	0.000	0.946	0.075 [0.067, 0.083]	0.046	−0.003	0.000	0.003
Invariance across											
ages	Configural	357.040	160.000	2.232	0.000	0.957	0.072 [0.063, 0.080]	0.038			
	Metric	376.150	170.000	2.213	0.000	0.954	0.071 [0.063, 0.080]	0.047	−0.002	0.000	0.009
	Scalar	399.040	180.000	2.217	0.000	0.952	0.071 [0.063, 0.079]	0.048	−0.002	0.000	0.001
Invariance across											
majors	Configural	400.350	160.000	2.502	0.000	0.950	0.077 [0.065, 0.084]	0.039			
	Metric	413.990	170.000	2.435	0.000	0.949	0.076 [0.068, 0.084]	0.049	−0.001	−0.001	0.010
	Scalar	436.600	180.000	2.426	0.000	0.947	0.075 [0.068, 0.083]	0.050	−0.002	−0.001	0.001

90% CI = 90% confidence interval.

### 4.3 Result based on the graded response model

Firstly, the parameter a presented in Table 7 represents the item discrimination, reflecting the ability of each item to differentiate between respondents with varying levels of the latent trait. The estimated discrimination parameters range from 1 to 3. Typically, discrimination values within the range of 0 to 2 are considered acceptable, while values exceeding 2 indicate excellent discrimination (Bulut and Desjardins, 2018). Accordingly, the results demonstrate that all items exhibit adequate discrimination, with items 2, 12, 17, 13, 26, and 15 displaying particularly strong discriminatory power. Of the five subconstructs of resilience, the perseverance dimension shows the strongest discriminative capacity, with all its items yielding discrimination parameters greater than 2.

**Table 7:** Item parameters estimated by the graded response model.

Item	a	b1	b2	b3	b4	b5	b6
item1	1.331 (0.114)	−5.036 (0.634)	−4.713 (0.540)	−3.725 (0.345)	−1.915 (0.162)	−1.246 (0.119)	0.787 (0.096)
item6	1.285 (0.108)	−5.180 (0.654)	−3.939 (0.369)	−2.849 (0.237)	−1.550 (0.139)	−0.718 (0.096)	1.072 (0.111)
item11	1.727 (0.129)	−4.304 (0.474)	−3.461 (0.290)	−2.606 (0.192)	−1.437 (0.112)	−0.639 (0.079)	0.966 (0.088)
item16	1.392 (0.112)	−4.900 (0.596)	−3.823 (0.349)	−2.835 (0.231)	−1.416 (0.125)	−0.647 (0.089)	1.095 (0.106)
item2	2.070 (0.137)	−3.337 (0.259)	−2.410 (0.159)	−1.380 (0.097)	−0.385 (0.066)	0.398 (0.066)	2.009 (0.134)
item12	2.058 (0.136)	−2.843 (0.196)	−2.149 (0.139)	−1.236 (0.090)	−0.114 (0.064)	0.829 (0.076)	2.236 (0.151)
item17	2.103 (0.138)	−3.033 (0.211)	−2.325 (0.147)	−1.411 (0.096)	−0.271 (0.064)	0.697 (0.072)	2.139 (0.143)
item13	2.722 (0.177)	−2.862 (0.184)	−2.242 (0.136)	−1.312 (0.085)	−0.249 (0.059)	0.501 (0.061)	1.853 (0.114)
item23	1.813 (0.127)	−3.134 (0.233)	−2.547 (0.178)	−1.455 (0.106)	−0.170 (0.067)	0.590 (0.074)	2.165 (0.155)
item26	2.536 (0.167)	−2.968 (0.200)	−2.285 (0.143)	−1.471 (0.093)	−0.396 (0.062)	0.342 (0.060)	1.723 (0.108)
item9	1.542 (0.113)	−3.766 (0.322)	−2.840 (0.212)	−1.801 (0.135)	−0.662 (0.083)	0.093 (0.072)	1.795 (0.139)
item14	1.791 (0.124)	−3.551 (0.293)	−2.497 (0.172)	−1.439 (0.106)	−0.281 (0.069)	0.425 (0.071)	2.144 (0.152)
item19	1.629 (0.118)	−3.648 (0.305)	−2.584 (0.186)	−1.727 (0.127)	−0.581 (0.078)	0.092 (0.070)	1.980 (0.147)
item5	1.978 (0.140)	−3.652 (0.315)	−2.876 (0.204)	−2.181 (0.145)	−1.238 (0.094)	−0.584 (0.072)	0.789 (0.077)
item10	1.467 (0.111)	−3.785 (0.324)	−2.995 (0.228)	−2.373 (0.177)	−1.111 (0.103)	−0.336 (0.078)	1.250 (0.110)
item15	2.714 (0.179)	−2.904 (0.201)	−2.353 (0.146)	−1.631 (0.100)	−0.682 (0.066)	−0.059 (0.058)	1.133 (0.078)

“a” means discrimination; “b1” to “b6” means step difficulty. Numbers in parentheses denote standard errors (SE).

Secondly, parameters b1 through b6 correspond to the category thresholds for the 7-point Likert scale utilized in this study. Ideally, these thresholds should span a suitable range to effectively capture variability across individuals with different resilience levels (Bulut and Desjardins, 2018). As shown in Table 7, for the value subscale (Items 1, 6, 11, and 16), the first thresholds (b1) are relatively low (−5.18 to −4.30), with Items 1 and 6 ≤ −5.00, indicating that the lowest response category is seldom endorsed.

In comparison, perseverance (b1 = −3.34 to −2.84; b6 = 2.01–2.24), emotion regulation (b1 = −3.13 to −2.86; b6 = 1.72–2.17), social support (b1 = −3.77 to −3.55; b6 = 1.80–2.14), and self-efficacy (b1 = −3.79 to −2.90; b6 = 0.79–1.25) cover students with higher level of resilience in learning English. Across items, thresholds are monotonically ordered, with step sizes of 0.6–1.4 logits, suggesting effective category functioning. Overall, the result of item discrimination and difficulty suggests that the scale’s parameters are generally appropriate, supporting its psychometric adequacy.

**Figure 3:**
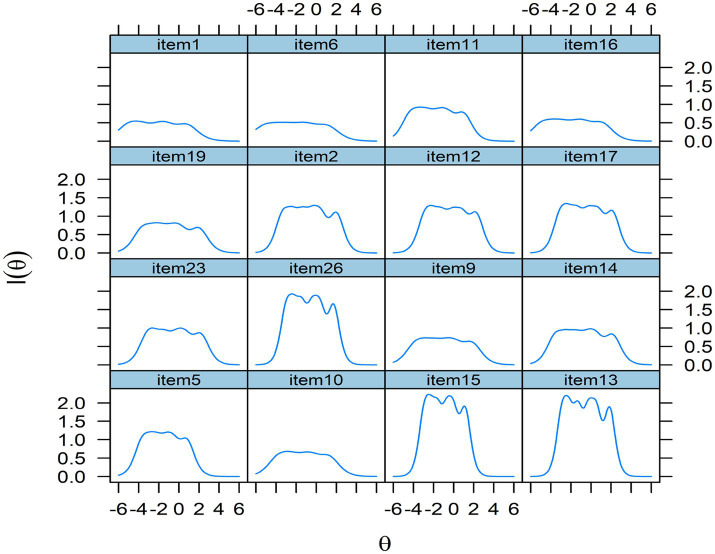
Item information function of 16 items.

Additionally, the item information function (see Figure 3) serves as a metric for assessing measurement precision across the latent trait continuum and offers valuable insight into the selection of items for the resilience scale. The peak of each function represents the point at which the item provides maximal information, with higher values reflecting greater precision at specific trait levels (Edelen and Reeve, 2007). As illustrated in Figure 3, items 13, 15, and 26 demonstrate the highest precision, indicating strong psychometric quality, whereas Item 1 provides the least information among the four items designed to measure the construct of value. Nevertheless, the findings suggest that the scale items are generally well-constructed and psychometrically robust, as none of the item information functions exhibited abnormal patterns.

**Figure 4:**
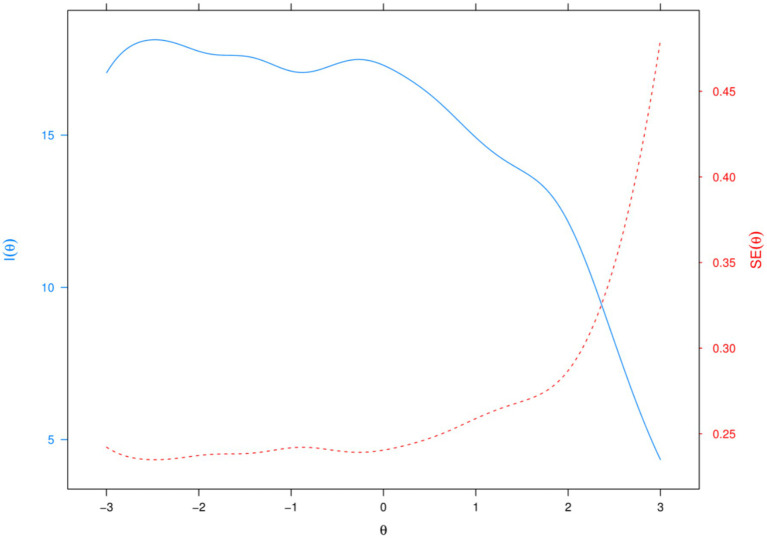
Measurement precision and error of the resilience scale.

Furthermore, Figure 4 presents the information function and corresponding measurement error curve of the English language learning resilience scale across varying levels of the latent trait (*θ*), representing the aggregated information from all items shown in Figure 3. The blue line indicates the amount of information provided by the scale, while the red dashed line represents the standard error of measurement. As shown in Figure 4, the scale provides the greatest amount of information for individuals with ability levels (θ) ranging from approximately −3 to 1.5, with the peak occurring around θ = −2. This result indicates that the scale achieves its highest measurement precision for learners with low to moderate levels of English language learning resilience. Such a pattern aligns well with the primary aim of this study, which is to identify and diagnose students who may be at risk of encountering persistent difficulties in English learning, thereby enabling timely and targeted pedagogical support. Overall, the psychometric properties reported above substantiated the reliability, validity, and robustness of the scale developed in this study.

## 5 Discussion

### 5.1 Discussion of factor structure

The factor structure of the resilience scale is crucial for understanding the underlying dimensions that the scale intends to measure. In this study, EFA and CFA were conducted to validate the resilience scale developed in this study.

The scree plot (see Figure 1) supported a five-factor solution, which collectively accounted for approximately 66% of the total variance, supporting the construct representation of the resilience scale. Additionally, examining individual factors, the value dimension explained the largest proportion of variance (approximately 15%), while the self-efficacy dimension accounted for the least (around 10%). The relatively lower variance explained by the self-efficacy factor may be influenced by measurement noise or the presence of cross-loadings. Future research could employ exploratory structural equation modeling (Shao et al., 2024) to more precisely estimate factor contributions and account for potential cross-loadings.

In addition, the CFA results demonstrated a good model fit, with strong convergent and discriminant validity supporting the five-factor model. These findings confirm the appropriateness of the selected factors and indicate that each factor contributes uniquely to the overall construct. This result further substantiates the construct validity of the resilience scale developed in this study.

Although several resilience scales have been previously developed and validated across various populations and contexts (e.g., Ricketts et al., 2015; Adıbelli et al., 2021; Nooripour et al., 2022; Cassidy, 2016), the current study notably differentiates itself by focusing on the development of a resilience scale specifically designed for Chinese EFL university students in the language learning context. This contextual specificity enhances the scale’s relevance and applicability to this target population.

Moreover, while Guo and Li (2022) have previously developed and validated a resilience scale aimed at English language learners, the present study advances this body of work by reconceptualizing the construct of resilience. This reconceptualization involved incorporating additional theoretically and empirically important constructs, such as learners’ perceived value of learning English and their self-efficacy (Wang et al., 2021), which are critical motivational and affective components influencing resilience in language learning. By integrating these constructs, the scale offers a more comprehensive and nuanced representation of resilience as it manifests in English language learning.

As reviewed in the literature section, resilience was defined based on a process approach (Chu et al., 2024; Fletcher and Sarkar, 2013), which covers how EFL learners are driven by the perceived value of English learning, engage in learning, persist through difficulties, and overcome challenges. These five factors represent different dimensions of resilience and are interrelated. To be specific, learners’ perceived value of learning English functions as the motivational foundation, fostering perseverance when facing challenges (Rivers, 2012). Upon encountering obstacles, learners engage in emotion regulation to manage negative affect (Zhang and Gao, 2024). If such regulation proves insufficient, they can seek social support to access external coping resources. These strategies contribute to the restoration or enhancement of EFL learners’ confidence (self-efficacy), which subsequently reinforces their overall resilience to tackle the challenges in the course of learning English. Overall, building on five theories reviewed in section 2.3, this study synthesizes these perspectives to reconceptualize the resilience construct in positive psychology. Accordingly, it advances our understanding of how resilience can be both theoretically grounded and empirically validated within the context of English language learning.

Nevertheless, the broadened and reconceptualized resilience model proposed in this study is by no means the endpoint of conceptualization. As noted by Fletcher and Sarkar (2013), resilience can be understood as a dynamic process of overcoming adversity and achieving positive adaptation. Accordingly, this study represents an initial validation effort that conceptualizes how students are motivated by the value of learning, regulated by their emotional control, and regain self-efficacy through seeking social support. Future research is encouraged to further refine and extend the conceptualization of resilience by incorporating other potentially important constructs.

### 5.2 Discussion of measurement invariance

The second research question investigated whether the scale is invariant across different genders, ages, and majors. The results indicated that the resilience scale showed both metric and scalar invariance across genders, ages, and majors. This is an encouraging finding in scale development and validation. As stressed by previous researchers (Kline, 2023), measurement invariance is critical for the generalizability of a scale, ensuring that scores can be interpreted appropriately, used fairly across groups, and that the scale demonstrates fairness-related validity.

The results discussed above are consistent with the meta-analysis on resilience and language learning achievement conducted by Chen et al. (2025). They reported that the proportion of female participants, grade level, and publication year were not significant moderators of the relationship between resilience and language learning achievement. This finding is also partially consistent with that of Duan et al. (2024), who found no significant gender differences in resilience but identified variations in resilience across different age levels.

The results of question two also fill in the gap of previous studies in the development and validation of the English language learning resilience scale. For instance, the resilience scale developed and validated by previous scholars (Qin et al., 2022) has not been fully investigated to ensure the robustness of the scale. The findings related to question two help bridge the gap in previous studies on the development and validation of resilience scales by providing positive evidence of measurement invariance across gender, age, and academic major.

Moreover, the results suggest that the scale possesses strong generalizability and can be applied across diverse groups of EFL learners in higher education. Accordingly, language educators and educational administrators can employ this tool to assess students’ resilience irrespective of gender, major, or age. Such assessment can, in turn, inform targeted interventions for students with lower resilience, enabling them to better cope with the challenges inherent in EFL learning and ultimately reducing the risk of burnout or dropout.

However, it is important to note that the applicability of this scale is currently limited to EFL learning in Chinese higher education contexts, as all participants in this study were EFL university students in China. Future studies could examine additional potential moderators of resilience. For example, Chen et al. (2025) reported that language type (e.g., Spanish) significantly moderated the relationship between resilience and learning achievement in their meta-analysis. Given that the current study focused solely on English language learners, future research should further explore whether the scale demonstrates comparable validity across other language learning contexts.

Additionally, given that cultural factors may influence how resilience is defined, expressed, and operationalized (Ungar, 2008; Motti-Stefanidi, 2018), the cross-cultural validity of the resilience construct measured by this scale remains largely unexplored. Future research could examine how the scale performs across different cultural and educational settings, including non-Chinese and bilingual populations, to better understand its broader applicability and potential for adaptation.

### 5.3 Discussion of IRT psychometric properties

The third research question aimed to validate the resilience scale using the graded response model. The results indicated that the items demonstrated strong discrimination and appropriate difficulty parameters, suggesting that they effectively differentiate individuals with varying levels of resilience. Additionally, the thresholds between response categories were well distributed, indicating that the scale offers meaningful measurement across a range of response levels.

Furthermore, the item information function indicated that the scale provides the highest measurement precision for students with low to average levels of resilience in English learning. This pattern contrasts with findings from Gao and Chen (2025), who reported that IRT-calibrated resilience scales in psychiatric contexts are most precise for individuals with high resilience. This difference highlights an important implication: the present scale is particularly well-suited for identifying learners who may be at greater risk due to lower resilience. By aligning measurement accuracy with educational needs, the scale offers practical value in diagnosing and supporting students who would benefit most from targeted interventions.

The results also indicated that items within the perseverance factor (items 2, 12, and 17) demonstrated the highest item discrimination and the greatest measurement precision among all five factors of resilience. One possible explanation is that this construct directly captures how Chinese university EFL learners cope with the challenges of English learning, which is considered a core component in the conceptualization of resilience (Fletcher and Sarkar, 2013).

Unexpectedly, the items within the value factor (excluding Item 11) exhibited the lowest discrimination power among all items across the five factors, although their factor loadings in EFA and CFA were above 0.7 (excluding Item 16). A plausible explanation is that English has been a compulsory subject in China from primary school through high school, and sometimes even at the university level. Consequently, most respondents perceive learning English as important, resulting in reduced variability and lower item discrimination. Another possible reason is that the association between value and resilience in English learning may be relatively weak, even though previous studies have shown that value is positively related to motivation (Kooken et al., 2015; Dörnyei, 1990).

Furthermore, the graded response model revealed a minor limitation in the resilience scale. As shown in Table 7, the step difficulties of item 1 from b1 to b2 ranged from −5.036 to −4.713, indicating a relatively narrow gap between these thresholds. This narrow spacing, corresponding to the “strongly disagree” and “disagree” categories, may slightly reduce the scale’s discriminant validity at the lower end of the ability spectrum, as it limits the instrument’s capacity to differentiate students with low resilience. Nevertheless, future studies could consider refining or retesting this item to further enhance the overall validity of the scale.

Despite this minor issue, the findings of question three also have important potential uses in second language teaching and learning. This study differs from previous studies (e.g., Liu et al., 2024) in resilience scale development by adopting the graded response model to further calibrate the robustness of resilience. The estimated item parameters (namely, item discrimination and step difficulties) can be used to build an adaptive testing system (Weiss and Sahin, 2024) to diagnose students’ resilience in learning English. In computer adaptive testing, the test can be either fixed-length or variable-length, which can enhance the practical value of the scale. Therefore, the items retained for resilience can be further utilized in practice. This can provide a system in which language teachers or educational administrators can timely monitor and evaluate the resilience state of students in learning English.

Overall, the finding of question three fills in the gap of previous studies (Ricketts et al., 2015; Turner et al., 2020) by providing the calibrated information based on the graded response model to put the scale into practical use, thereby enhancing its real-world applicability.

## 6 Implications

Building on the previous resilience scale (Liu et al., 2024; DeSimone et al., 2017), this study has validated a five-factor (value, perseverance, emotion regulation, social support, and self-efficacy) resilience scale in English language learning, which has important theoretical, pedagogical, and practical implications.

Theoretically, drawing on the five foundational theories reviewed in the literature, this study adopts a process-oriented approach to reconceptualize resilience within the framework of positive psychology. In contrast to previous English language learning scales (e.g., Guo and Li, 2022), which focus on a limited set of dimensions, the scale developed and validated here expands the construct representation by incorporating previously overlooked components such as emotional regulation and perseverance. This enhanced reconceptualization not only offers a more nuanced and comprehensive operationalization of resilience but also provides a clearer theoretical foundation for understanding how resilience can be defined, measured, and applied in the context of second language learning.

Pedagogically, the multidimensional resilience constructs can encourage language teachers to pay extra attention to students’ resilience conditions in language learning rather than solely focusing on developing students’ linguistic ability. The resilience framework can shed light on how to develop a supportive language learning environment, enhancing students’ learning motivations and resilience. Language teachers can also use the validated resilience scale to measure students’ resilience, based on which language teachers can provide different remedial support.

Practically, the validated resilience scale can serve as a reliable diagnostic instrument for related stakeholders (e.g., teachers, school administrators, and government) to monitor students’ resilience in language learning. Educational institutions can adopt this scale to provide early diagnostic feedback and allocate corresponding resources to help those students in need. Additionally, language learners can also use this scale as a self-assessment tool to raise their awareness of resilience, promoting their autonomy and psychological well-being in language learning.

## 7 Conclusion

This study set out to develop and validate the English language learning resilience scale, addressing a gap in existing resilience measurement tools by tailoring the instrument to the specific context of English learning among Chinese EFL university students. Results from EFA and CFA confirmed the multidimensional nature of the resilience construct, comprising five distinct factors—perceived value of learning, perseverance, emotion regulation, social support, and self-efficacy—with satisfactory reliability and strong convergent and discriminant validity. Multi-group CFA demonstrated robust scalar measurement invariance across gender, academic major, and age, while graded response model analysis revealed strong item discrimination and appropriate difficulty parameters, with the highest measurement precision for learners exhibiting lower resilience levels. Collectively, these psychometric properties provide compelling evidence that the scale is a reliable, valid, and robust diagnostic tool for assessing resilience in English language learning.

Nevertheless, several limitations should be acknowledged. First, the sample size from only two universities may constrain the generalizability of the findings. Future research could address this by recruiting a larger and more diverse sample, including students from multiple universities and a wider range of language-related majors, to enhance the applicability of the scale. Second, the present study primarily examined the effects of gender and academic major on EFL learners’ resilience. Future investigations may extend this line of inquiry by exploring additional variables—such as learners’ language proficiency levels and diverse cultural backgrounds—to gain a more comprehensive understanding of the factors shaping resilience in English language learning. Finally, this study validated the scale solely through psychometric analyses of questionnaire data, representing an initial validation effort. The external validity of the scale, based on other sources of evidence (e.g., learning outcomes), warrants further investigation to provide more comprehensive support for its validity.

## Data Availability

The raw data supporting the conclusions of this article will be made available by the authors, without undue reservation.

## Appendix

**Table A1:** Journal article reporting standards checklist.

Section	Description	Location in manuscript
Introduction	Importance of resilience in second language learning Rationale for developing a new resilience scale	Introduction
Literature review	Conceptualization of resilience Selection and theoretical grounding of the five resilience factors	Sections 2.2 and 2.3
Measures/instruments	5-point Likert scale 25-item English Language Learning Resilience Scale	Methods, Section 3.1
Sampling and data clearning	Participants recruited from two Chinese universities (valid *N* = 617) Age and gender reported Data clearning documented •Reduced to 16 items after item analysis	Methods, Section 3.2
Ethics statement	Ethical approval obtained from the universities of the first and third authors Oral informed consent obtained from participants	Methods, beginning of Section 3.2
Design/procedure	Cross-sectional survey design Demographic information collected Ethical clearance secured	Methods, Section 3.2
Data analysis	Exploratory Factor Analysis (EFA), Confirmatory Factor Analysis (CFA), and Graded Response Model (GRM) conducted Software used: SmartPLS 4 and R (packages: SPSS, mirt, careless); Version of software reported	Methods, Section 3.3
Results	Factor loadings from EFA and CFA reported Model fit indices presented Measurement invariance tested Parameters estimated using graded response model Standard errors and confidence intervals provided	Results, Sections 4.1–4.3
Discussion – interpretation	Interpretation of the five-factor structure Comparison with previous research Highlights of the study’s unique contributions	Discussion, Sections 5.1–5.3
Discussion – generalizability	Discussion of the scale’s applicability across populations and cultural contexts	Discussion, end of Section 5.2
Implications	Theoretical, pedagogical, and practical implications discussed	Section 6.1
